# Buffalo Medical and Surgical Association

**Published:** 1884-03

**Authors:** Clayton M. Daniels

**Affiliations:** Secretary


*Secretary.*

---

### BUFFALO MEDICAL AND SURGICAL ASSOCIATION.

*Regular Meeting, February 5th, 1884.*

Vice-President Dr. F. W. Bartlett in the Chair, Dr. C. M. Daniels, Secretary.

Present—Drs. Hartwig, Coakley, Burkhardt, Frederick, Weil, F. H. Potter, Wyckoff, Hubbell, and by invitation Dr. Keene.

Application for membership received from Dr. Mary Berkes who was duly elected.

Essay by Dr. C. C. Frederick, subject, "Spinal Irritation."

*Discussion*—Dr. Hartwig said he felt amply repaid for coming to hear the paper and that it deserved due credit. Thought there was considerable uncertainty regarding the expression, "spinal irritation." It had been more commonly used in the past than at present, and he thought "neurasthenia" should be substituted. Considered that great doubt existed in so complex a subject and that we should endeavor to look up the pathological entities and divide the group into its several divisions.

Dr. Bartlett considered the subject very interesting, although the term "spinal irritation" was very indefinite. He agreed with Dr. Hartwig in relation to its division. Reported a case wherethe extreme irritation was clearly traced to excessive use of tobacco. Thought that to get at the cause was the all important point. Also spoke of the giving of quinine in very large doses in cases of supposed malarial origin, which practice he condemned.

Dr. Frederick in closing said he spoke of this condition as not belonging to the true term, and that in fact "anemia" of itself was the point and not "spinal irritation" *per se.*

*Voluntary Communications*—Dr. Burkhardt read an original article on "A Medical View on Clothing and its Fashion." It was essentially a comparative view with the natural covering of the lower animals and argued ably and favorably for a more judicious mode of dress, especially about the *back*, which was readily shown to be the least protected and all apparently on account of the fashion of forming garments for *show*, in front, (as the vest) and the back made of thinest material.

The writer referred to the possibility of such exposures as being the primary cause of various diseases, as albuminuria, &c., where certain organs were called upon to bear more than a proportionate amount of cold.

Dr. Hartwig reported case of a man who for two years had his feet frequently exposed to an excessive heat while standing upon a heated boiler. In August last he noticed a sudden dimness of sight of right eye. This improved a little. Right arm soon became very weak, nearly paralytic, right leg soon followed. Improvement gradually occurred, then the left side became involved to a certain extent. Mental faculties perfect. General improvement continues slowly.

Dr. H. made the report to obtain a diagnosis from the members present.

Dr. Bartlett said that conditions of this kind showed movable or transitional engorgements of spine of central origin. Also reported a case of great "Alopecia" where there was no apparent cause, not one single hair being discoverable upon any portion of the body.

Dr. Coakley asked the treatment in the case reported by Dr. Hartwig.

Dr. Hartwig replied that he had tried pot. iodide, ergotine, rest, galvanism, etc., but could not say that either was of much value.

Dr. Wyckoff read a very interesting report of a case of rupture of the heart (given in full in the JOURNAL for February).

Dr. Bartlett, in commenting upon the case, considered it very rare and important.

*Prevailing Diseases*—Scarlet fever with tendency to croup, measles.

Adjourned. CLAYTON M. DANIELS,
*Secretary.*

